# Supplementary material for: Traumatic dislocation of middle ear ossicles: A new computed tomography classification predicting hearing outcome
Source: PLoS One. 2021 Feb 8;16(2):e0245796. doi: 10.1371/journal.pone.0245796 (PMC7870152; doi:10.1371/journal.pone.0245796)
Supplement: S1 Appendix — (PDF) [file pone.0245796.s001.pdf]

Appendix

Table S1. Inter-Rater Agreement for Ossicular Chain Luxations

Parameter Assessed by Radiologist n=68	Agreement Cohen’s Kappa/ Intraclass Correlation Coefficient	*P* Value
Detection of luxation/dislocation (no, incudostapedial, incudomalleolar, stapedo-vestibular, complex)	0.769	<.001
Incus axis deviation (no, medial, lateral)	0.156	.06
Distance between ossicles (yes,no)	0.760	<.001
Ossicular joint space (normal, hemorrhage, air)	0.696	<.001
Malleus-incus axis distance ‘D’ (mm)	0.817	<.001
Malleus-incus axis angle ‘β’ (deg)	0.825	<.001

Table S2. Normative Data for Incudomalleolar Joint[a]

Parameter, n=34	Min.	Max.	Mean	SD	Mean ± 2 SD	
Malleus-incus axis distance ‘D’ (mm)	0.0	0.2	0.006	0.03	0	0.07
Malleus-incus axis angle measured at midpoints ‘α’ (deg)	0.0	2.5	0.074	0.42	0	0.91
Malleus-incus axis angle ‘β’ (deg)	55.4	90.9	74.9	9.18	56.5	93.2
Ossicle joint space ‘d’(mm)	0.0	1.0	0.676	0.26	0	1.19

[a]contralateral healthy ear

Figure S1. Flow diagram showing the procedure for patient selection and analysis.

Abbreviations: CT computed tomography.

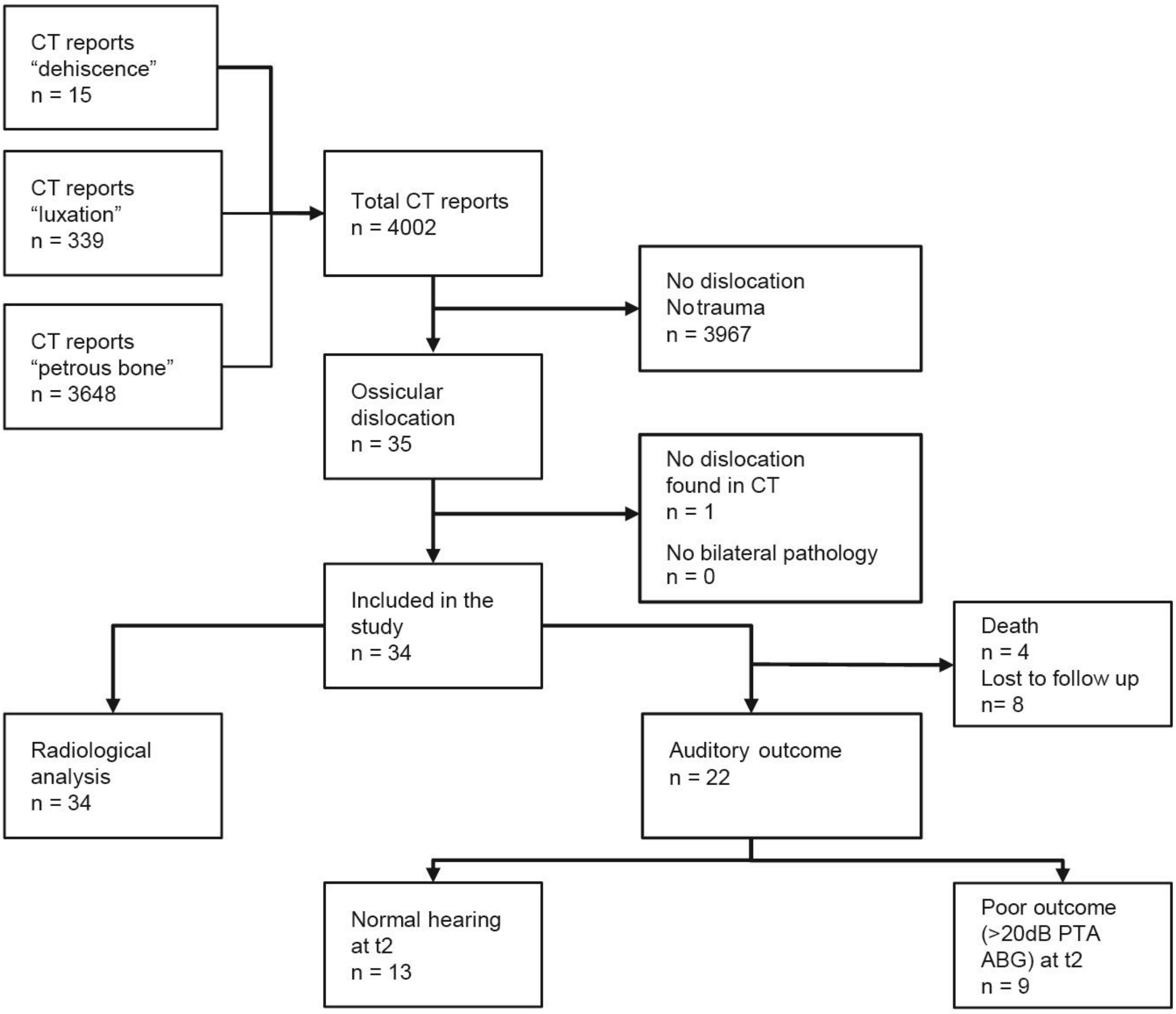

Figure S2. Incudal ligament folds

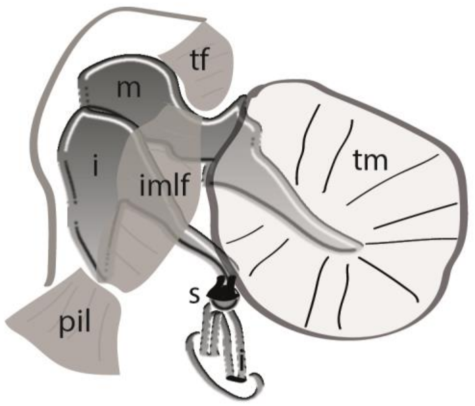

pil: posterior incudal ligament, imlf: incudomalleolar ligament fold, tf: tensor fold, tm: tympanic membrane, i: incus, s: stapes, m: malleus
